# Differential localizations of protein phosphatase 1 isoforms determine their physiological function in the heart

**DOI:** 10.1093/abbs/gmy171

**Published:** 2019-02-05

**Authors:** Ruijie Liu, Christian Miller, Christiana D’Annibale, Kimberly Vo, Ashley Jacobs

**Affiliations:** Department of Biomedical Sciences, Grand Valley State University, Allendale, MI, USA

**Keywords:** cardiac function, histone deacetylase, phospholamban, protein phosphatase 1

## Abstract

Protein phosphatase 1 isoforms α, β, and γ (PP1α, PP1β, and PP1γ) are highly homologous in the catalytic domains but have distinct subcellular localizations. In this study, we utilized both primary cell culture and knockout mice to investigate the isoform-specific roles of PP1s in the heart. In both neonatal and adult cardiac myocytes, PP1β was mainly localized in the nucleus, compared to the predominant presence of PP1α and PP1γ in the cytoplasm. Adenovirus-mediated overexpression of PP1α led to decreased phosphorylation of phospholamban, which was not influenced by overexpression of either PP1β or PP1γ. Interestingly, only cardiac-specific knockout of PP1β resulted in increased HDAC7 phosphorylation, consistent with the predominant nuclear localization of PP1β. Functionally, deletion of either PP1 isoform resulted in reduced fractional shortening in aging mice, however only PP1β deletion resulted in interstitial fibrosis in mice as early as 3 weeks of age. Deletion of neither PP1 isoform had any effect on pathological cardiac hypertrophy induced by 2 weeks of pressure overload stimulation. Together, our data suggest that PP1 isoforms have differential localizations to regulate the phosphorylation of their specific substrates for the physiological function in the heart.

## Introduction

The balance between protein phosphorylation and dephosphorylation regulates almost every aspect of biological processes [1]. In contrast to protein phosphorylation mediated by a vast number of protein kinases, only a small number of protein phosphatases have been reported [2]. Protein phosphatases are classified into three subgroups, namely serine/threonine, tyrosine, or dual specific phosphatases [3]. The serine/threonine protein phosphatases, which include protein phosphatase 1 (PP1), protein phosphatase 2A (PP2A), and protein phosphatase 2B (PP2B, also called calcineurin), account for ~98% of the phosphatase activities in the mammalian heart [4]. A common feature for the serine/threonine phosphatases is that they are holoenzymes composed of both catalytic subunits for the enzymatic activity, and regulatory subunits that are either targeting proteins or substrates [4]. The three catalytic subunits of PP1s (PP1α, PP1β, and PP1γ), encoded by three distinct genes, are highly homologous in their catalytic domains. Their divergent N- and C-termini are hypothesized to underlie the target selectivity [5–7].

Sympathetic stimulation of cardiac myocytes generates cAMP which in turn activates protein kinase A (PKA). PKA phosphorylates many Ca^2+^ handling and myofilament proteins involved in cardiac contraction, such as ryanodine receptor 2 (RyR2), phospholamban (PLB), troponin I (TnI), myosin light chain 2 V (MLC2V), and myosin binding protein C [8–13]. Phosphorylation of sarcoplasmic reticulum (SR) protein PLB at serine 16 by PKA or threonine 17 by calcium-calmodulin-dependent protein kinases (CAMKs) increases the ATPase activity of SR Ca^2+^ transport ATPase (SERCA) to enhance Ca^2+^ reuptake into the SR [14]. In heart failure patients, PP1 activities were found to be increased, suggesting that PP1s might be the therapeutic targets for heart failure. Consistent with this notion, adeno-associated virus (AAV) 9-mediated overexpression of phosphatase 1 inhibitor-1 (I-1) improved cardiac function, while overexpression of a catalytic PP1α isoform in mice led to reduced cardiac function and heart failure [15,16]. However, cardiac-specific deletion of PP1β led to heart failure and cardiac remodeling in mice [17]. These findings further extended the functional complexity of PP1 isoforms in the heart, possibly due to the uncharacterized roles of PP1s in other subcellular locations such as the nucleus [17–19].

Histone deacetylases (HDACs) are a group of enzymes that remove the acetyl groups from lysine residues of a variety of target proteins including histone and non-histone substrates [20,21]. In mammalian cells, 18 HDACs have been identified and grouped into five different subgroups I, IIa, IIb, III, and IV. In the heart, group IIa HDACs (HDAC4, HDAC5, HDAC7, and HDAC9) have been extensively studied and shown to be the endogenous inhibitors for cardiac hypertrophy [20]. For example, overexpression of HDAC4, HDAC5, and HDAC9 suppressed myocyte enhancer factor 2 mediated gene expression to prevent pathological stimuli-induced cardiac hypertrophy [22–24]. Loss of either HDAC5 or HDAC9 led to cardiac hypertrophy in mice [23,25]. Moreover, class II HDACs are transcriptional repressors at resting state. Upon stimulation, phosphorylation of class II HDACs led to their nuclear export, resulting in the de-repression of their targeting genes [19,26–28]. However, the exact protein phosphatases that regulate the phosphorylation of HDACs have yet to be explored.

In the present study, we investigated the subcellular localization of three PP1 isoforms and determined their specific substrates. In addition, we determined whether PP1s play a role in physiological and pathological heart function utilizing knockout mice with cardiac-specific deletion of each isoform.

## Materials and Methods

### Mice husbandry and transverse aortic constriction surgery

All animal experimentation was approved by the Institutional Animal Care and Use Committee at Grand Valley State University (Protocol Number: 17-02-A). Mice used in this study were initially generated in Dr. Molkentin’s laboratory at Cincinnati Children’s Hospital (Cincinnati, USA) [17], and housed in standard rodent cages, and observed daily for their wellbeing such as physical activity and food/water intake. To achieve cardiac-specific deletion of PP1 isoforms, mice were targeted with Cre recombinase-dependent LoxP sequences flanking exon 3 of each PP1 gene as described previously [17]. These mice were subsequently crossed with NKX2.5-Cre knock-in mice (030047; The Jackson Laboratory, Bar Harbor, USA) to achieve PP1 deletion since early development [17]. Mice were viable and had similar lifespan between all four groups (NKX-Cre, PP1α fl/fl^NKX-Cre^, PP1β fl/fl^NKX-Cre^, and PP1γ fl/fl^NKX-Cre^), except PP1β deletion mice which developed cardiac dysfunction as early as 3 weeks of age. For mouse aging study, sex-matched mice were analyzed at 2, 4, and 8 months of age by echocardiography for the fractional shortening and other ventricular parameters.

The procedures for pressure overload by transverse aortic constriction (TAC) to induce cardiac hypertrophy have been previously described [17]. In brief, 8-week-old sex-matched mice were anesthetized by 1.5% isoflurane to expose the transverse aortas through a thoracotomy procedure. Constriction was performed with 7–0 silk (Ethicon, Somerville, USA) around a ~27-gauge wire which was slowly removed after the constriction. After the surgery, mice were given pain medicine buprenex (0.1 mg/kg), and transferred to 30°C chambers for overnight recovery before being transferred back to the standard housing. After 2 weeks of TAC, Doppler echocardiography was first applied to the mice to determine pressure gradients across the constrictions to ensure similar stimulation for all the mice. Cardiac function of the mice was analyzed by echocardiography using a SONOS 5500 instrument (Hewlett-Packard, Palo Alto, USA) and a 15-MHz transducer. Left ventricular fractional shortening percentage (FS) was calculated using left ventricle internal diameters at the end of systole (LVIDs) and diastole (LVIDd) based on the following formula: FS (%) = [(LVIDd−LVIDs)/LVIDd] × 100%. At the end of the echocardiographic analysis, mice were euthanized by CO_2_ inhalation for 5 min followed by cervical dislocation before the hearts were removed for the weight measurement. Hearts were then fixed overnight in 10% formalin-containing phosphate-buffered saline (PBS) and dehydrated for embedding with paraffin. Serial 5-μm heart sections were stained with Masson’s trichrome to detect interstitial fibrosis shown as the blue staining. Fibrosis was quantified as percentage of blue staining of the tissue using ImageJ.

### Isolation of cardiomyocytes and adenovirus infection

Neonatal rat hearts were digested with 0.01% trypsin (code TRLS; Worthington, Lakewood, USA) in sterile calcium and magnesium-free Hank’s Balanced Salt Solution (HBSS) for 16–20 h at 4°C with gentle rotation. The next day, trypsin inhibitors (code SIC; Worthington) were added to the digestion tube and incubated for 15 min at 37**°**C to stop the trypsin activity. Collagenases of 1500 units (code CLSPA; Worthington) were then added to the digestion tube for an additional digestion for 60 min. Cells were collected through a cell strainer, and plated into 0.01% gelatin-coated dishes with 5% fetal bovine serum containing medium M199 (10-060-CV; Corning, Corning, USA). After 60 min of culture, myocytes were washed off the plates and seeded into new gelatin-coated dishes, while the cells attached to the plates were considered as non-myocytes/fibroblasts. For adenovirus infection, neonatal myocytes were first switched to serum-free medium and then incubated with purified adenoviruses expressing either β-galactosidase [29] or PP1α (SL112646; SignaGen Laboratories, Rockville, USA), PP1β (SL112778; SignaGen Laboratories), PP1γ (SL112648; SignaGen Laboratories) for 2 h to enhance infection efficiency. Later, 10% serum containing medium was added to the myocytes for a total infection of 36 h. To analyze the protein phosphorylation status influenced by PP1 expression, myocytes were first serum starved for 30 min in serum-free medium, then stimulated with 10 μM isoproterenol (16504; Sigma, St Louis, USA) for 5 min, and harvested into lysis buffer containing 20 mM HEPES, 150 mM NaCl, 1% Triton X-100, 1 mM EDTA, and protease/phosphatase inhibitors. Proteins were harvested after centrifugation, and after concentration quantification, protein samples (10 μg) were loaded for the western blot analysis. The primary antibodies against the following proteins were used: p^Ser16^-phospholamban (A010-12AP; Badrilla, Leeds, UK), p^Thr17^-phospholamban (A010-13AP; Badrilla), phospholamban (MA3-922; Thermo Fisher, Waltham, USA), and GAPDH (10–1500; Fitzgerald, Acton, USA).

Isolation of adult ventricular myocytes has been described previously [30]. Hearts were surgically removed from 2-month-old mice after treatment with heparin (0.35 units) under anesthesia (Nembutal, 100 mg/kg), and cannulated for retrograde perfusion with a solution containing liberase blendzyme (05401151001; Roche, Indianapolis, USA). Heart tissues were dissociated by gentle pipetting and filtered into a new tube through a 500-μm mesh to eliminate the non-digested tissues. Myocytes were allowed to settle by gravity followed by CaCl_2_ re-introduction. The cells in the pellet were considered as myocytes, while those in the supernatant were considered as non-myocytes/fibroblasts. Small aliquots of isolated cells were used to count rod-shaped cardiac myocytes using a hemocytometer (02-671-6; Fisher Scientific, Pittsburgh, USA). A high proportion (>80%) of rod-like cells were used for the following RNA and immunofluorescence analysis.

### RNA isolation and real-time PCR

Total RNA was isolated from myocytes and fibroblasts using the RNeasy Fibrous Tissue Kit (74704; Qiagen, Hilden, Germany) and quantified with a NANODROP 2000 Spectrophotometer (Thermo Scientific). cDNA synthesis was performed using the SuperScript III First-Strand Synthesis Kit (18080-051; Invitrogen, Carlsbad, USA) on a regular PCR machine. Real-time PCR analysis was performed using SYBR green dye (172–5274; Bio-Rad, Hercules, USA) on a CFX96 real-time PCR detection system (Bio-Rad). The primers used to determine the mRNA level of PP1s were as follows: PP1α, forward: 5′-cctccagagagcaactacctcttc-3′, reverse: 5′-acgtcttccacagtttgatgttgt-3′; PP1β, forward: 5′-aatatggaggttttccaccagaag-3′, reverse: 5′-attgatgctagcacactcatggtt-3′; PP1γ, forward: 5′-tcttcctcagtcagcctatccttt-3′, reverse: 5′-ctccggatacttgattttgtaggc-3′; and ribosomal protein 27 as the internal control, forward: 5′-ggacgctactccggacgcaaag-3′, reverse: 5′-cttcttgcccatggcagctgtcac-3′.

### Nuclear fractionation and western blot analysis

Procedures for nuclear fractionation of proteins from a cell culture have been previously described [30]. In brief, neonatal or adult rat cardiac myocytes were collected into hypotonic buffer A (10 mM HEPES, pH 7.9, 10 mM KCl, 0.1 mM EDTA, 0.4% IGEPAL, and protease inhibitors), and were then lysed on ice for 30 min. After centrifugation for 5 min at 15,000 *g*, the supernatant was collected as cytosolic fraction and the pellet was further re-suspended into buffer B (20 mM HEPES, pH 7.9, 0.4 M NaCl, 1 mM EDTA, 10% glycerol, and protease inhibitors). After 2 h of incubation on ice, proteins eluted from the pellet were collected by centrifugation and considered as nuclear proteins. After protein concentration quantification, 40 μg of cytosolic and nuclear proteins were loaded and separated by 10% SDS-PAGE, transferred onto PVDF membrane, blocked with 5% non-fat milk, and incubated with the following primary antibodies: anti-PP1α antibody (sc-6104; Santa Cruz Biotechnology, Dallas, USA), anti-PP1β antibody (07-1217; Millipore, Burlington, USA), anti-PP1γ antibody (sc-6108; Santa Cruz Biotechnology), anti-GAPDH antibody (10-1500; Fitzgerald), anti-Lamin A/C antibody (2032; Cell Signaling Technology, Danvers, USA). Goat-anti-rabbit secondary antibody (P/N 925-32211; Li-COR, Lincoln, USA) and goat-anti-mouse secondary antibody (P/N 925-32210; Li-COR) were utilized to visualize the results by the Licor-Odyssey system. Additional primary antibodies against HDAC7 (sc-74563; Santa Cruz Biotechnology) and phospho-HDAC7 (3424; Cell Signaling Technology) were used for the western blot analysis on heart tissues. Phosphorylation of MLC2 was detected by a ProQ-Diamond staining method as previously described [17].

### Immunocytochemistry

Rat cardiac myocytes grown on laminin or gelatin-coated slides were fixed with 4% paraformaldehyde for 20 min followed by rinses with PBS for three times. Cells were permeabilized with 0.3% Triton X-100 for 15 min, then blocked with 3% bovine serum albumin for 20 min. Isoform-specific anti-PP1 antibodies or together with anti-sarcomeric alpha-actinin antibody (A7732; Sigma, St Louis, USA) were used for overnight staining, which were then detected by Alexa Fluor 488 or Alexa Fluor 594 conjugated secondary antibody (A11032 and A11034; Thermo Fisher). DAPI (D1306; Thermo Fisher) was used for counterstaining of the nucleus, and all the images were taken using Nikon A1 confocal microscope (Tokyo, Japan). Quantification of the relative fluorescence intensity of PP1 staining in the neonatal cardiac myocytes was performed by FIJI-ImageJ.

### Statistical analysis

All the results were presented as the mean ± SEM. Statistical analysis was performed using Microsoft Excel using Student’s *t*-test for two group analysis or ANOVA followed by a Bonferroni *post hoc* test for comparison of differences across multiple groups. *P* < 0.05 was considered statistically significant.

## Results

### Distinct subcellular localizations of PP1 isoforms

**Figure 1.**
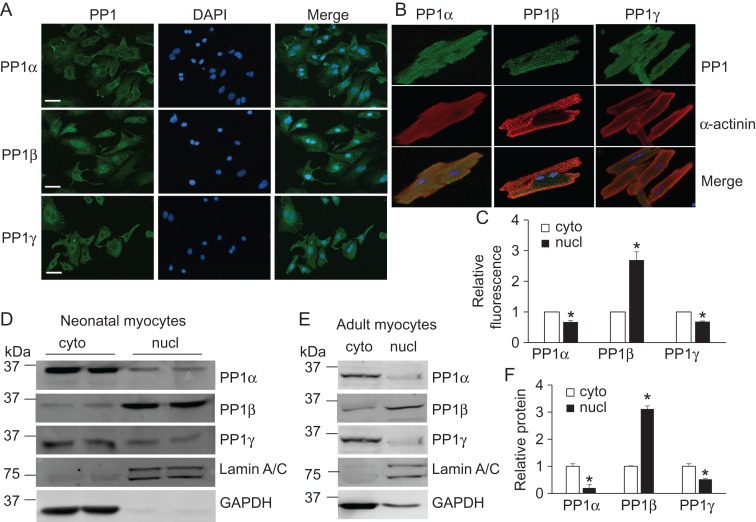
**Distinct subcellular localizations of PP1 isoforms in cardiac myocytes** (A) Immunocytochemistry analysis of endogenous PP1 isoforms in neonatal rat cardiac myocytes. Scale bar, 10 μm. (B) Immunocytochemistry analysis of endogenous PP1 isoforms in adult rat cardiac myocytes. Magnification, 400×. (C) Quantification of relative fluorescence intensity of PP1 isoforms based on A using FIJI-ImageJ. *P < 0.05 vs cyto. Western blot analysis of PP1s in the cytoplasm (cyto) and nucleus (nucl) of neonatal (D) and adult (E) rat cardiac myocytes. Lamin A/C and GAPDH were used as controls for nuclear and cytosolic proteins respectively. (F) Quantification of PP1 isoforms based on E. *P < 0.05 vs cyto. All the experiments were repeated three times with similar results.

PP1 catalytic isoforms have been demonstrated to differentially regulate the phosphorylation of myofilament proteins including myosin light chain 2 (MLC2) and myosin binding protein C [17]. However, little is known whether they are targeted to other subcellular locations to potentially regulate cardiac function. To address this question, we compared the localizations of endogenous PP1s in both neonatal and adult cardiac myocytes using isoform-specific antibodies we previously identified [17]. The majorities of the PP1α and PP1γ proteins in neonatal cells were in the cytoplasm compared to their nuclear localization (100% versus 67%) (**Fig. 1A,C**). PP1β was predominantly present in the nucleus (2.7 times more) (**Fig. 1A,C**). We also assessed their localization in isolated adult rat cardiac myocytes which were fully differentiated. Compared to the absence of PP1α and PP1γ in the nucleus, PP1β was also found in the nucleus although not as obvious as in the neonatal cardiomyocytes (**Fig. 1B**). To further biochemically confirm this data, we performed a crude nuclear fractionation experiment to study the relative distribution of each PP1 isoform. In both neonatal and adult cells, PP1β was predominantly present in the nucleus compared to PP1α and PP1γ that had a higher expression in the cytoplasm (**Fig. 1D–F**). These data showed that PP1s have different localization, which indicates that PP1s might regulate distinct targets due to their specific localization.

### PP1α and PP1β differently regulate substrate phosphorylation in the heart

**Figure 2.**
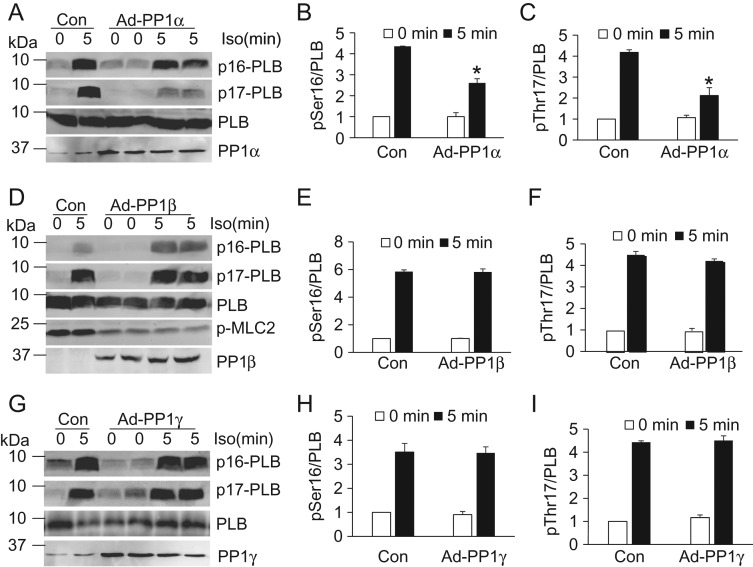
**PP1s differentially regulate substrate phosphorylation in neonatal cardiac myocytes** Western blot analysis for the phosphorylation of PLB at serine 16 and threonine 17, total PLB, and PP1s from neonatal rat cardiac myocytes infected with adenoviruses expressing either β-gal (Con) or individual PP1 isoform. p-MLC2 was performed using ProQ-Diamond staining method. Quantification was based on individual western blot. *N* = 4 for each group. *P < 0.05 vs. Con 5 min.

**Figure 3.**
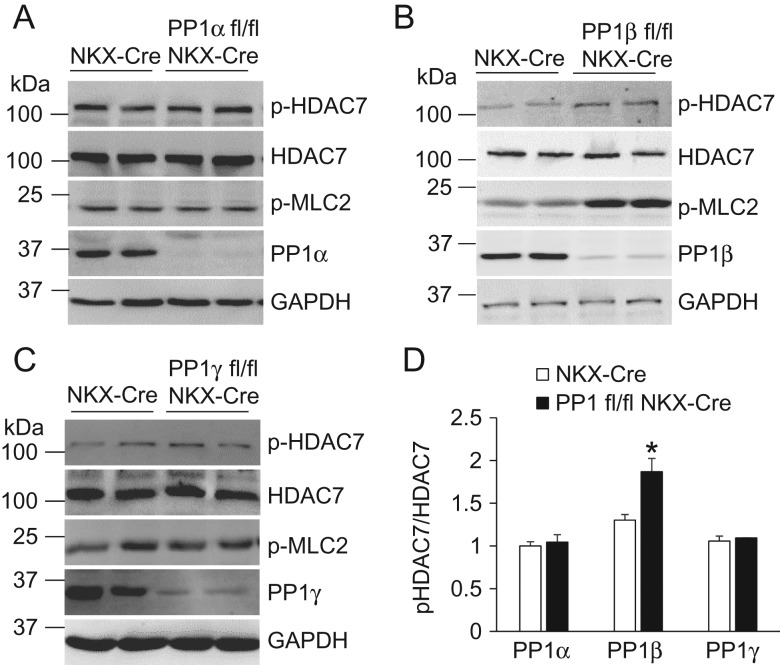
**Deletion of PP1β from mouse heart leads to increased HDAC7 phosphorylation** (A–C) Western blot analysis for phospho-HDAC7, HDAC7, PP1α, PP1β, PP1γ, and GAPDH from the hearts of the indicated mice at 2 months of age. Phosphorylation of MLC2 was detected using a ProQ-Diamond staining method [17]. (D) Quantification of western blots shown in A–C. *N* = 4 for each group. *P < 0.05 vs. NKX-Cre.

Based on the subcellular localization of PP1 isoforms (**Fig. 1**), we further investigated whether they preferentially dephosphorylate substrates. Previous studies using either RNAi or knockout mice have indicated PLB and MLC2V as the potential substrates [17,18]. Here, we used a gain-of-function approach to assess the phosphorylation of these targets by adenovirus-mediated overexpression of each PP1 isoform in neonatal cardiac myocytes. Due to the low yield and survival during culture, adult myocytes were not chosen for the biochemistry analysis. Overexpression of PP1α significantly reduced the phosphorylation of PLB in cells challenged with isoproterenol (**Fig. 2A–C**). However, overexpression of either PP1β or PP1γ did not alter the phosphorylation of PLB (**Fig. 2D–I**). These data suggest that PP1α but not PP1γ, the PP1 isoform localized in the cytoplasm, regulates the phosphorylation of PLB in the cytoplasm. Only expression of PP1β reduced the phosphorylation of MLC2, consistent with the increased MLC2 phosphorylation upon knockout of PP1β from the mouse heart (**Fig. 2D**) [17]. Because PP1β was also enriched in the nucleus (**Fig. 1**), we sought to identify the nuclear substrate for PP1β. As PP1β is involved in HDAC7 dephosphorylation in thymocytes [19], we first assessed HDAC7 using cardiac-specific PP1 deletion mice (PP1α fl/fl^NKX-Cre^, PP1β fl/fl^NKX-Cre^, and PP1γ fl/fl^NKX-Cre^ respectively) [17]. Compared to the NKX-Cre mice, deletion of endogenous PP1β, but not PP1α or PP1γ, significantly enhanced the phosphorylation of HDAC7 (**Fig. 3**). Consistent with our previous study, the phosphorylation of the myofilament protein MLC2 was increased in PP1β deletion hearts (**Fig. 3B**). Together, these data showed that PP1s regulate different substrates, indicating they might have different functional roles in the heart.

### PP1s are essential for physiological cardiac function

**Figure 4.**
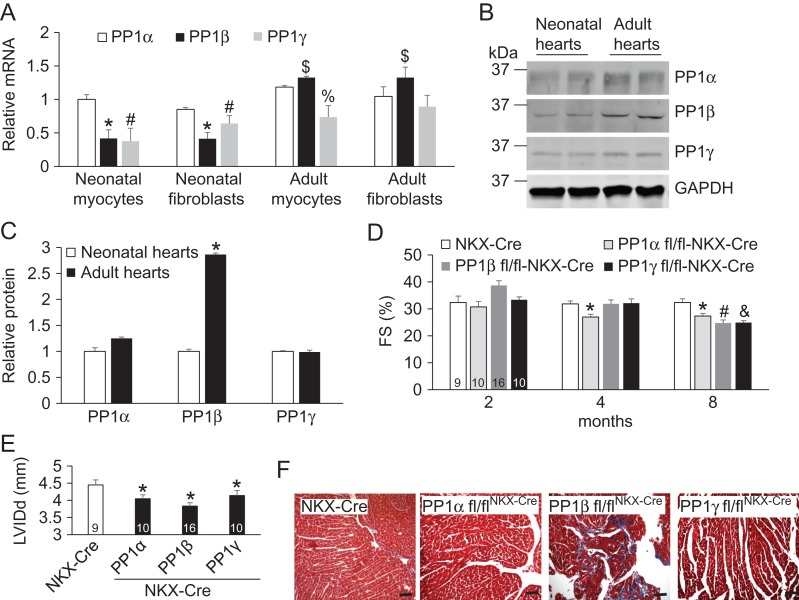
**PP1s are essential for the physiological cardiac function** (A) Real-time PCR analysis of PP1 expression in cardiac myocytes and fibroblasts isolated from neonatal and adult mouse hearts. *N* = 4 for each group. *P < 0.05 vs PP1α from neonatal cells; ^#^*P* < 0.05 vs PP1α from neonatal cells; ^$^*P* < 0.05 vs PP1α from adult cells; ^%^*P* < 0.05 vs PP1α from adult myocytes. (B) Western blot analysis for each PP1 isoform in the neonatal and adult mouse hearts. (C) Quantification of protein levels of PP1s in B. *N* = 4 for each group. *P < 0.05 vs neonatal hearts. (D,E) Fractional shortening (FS) and LVIDd of the aging mice. Mouse numbers for four groups were indicated in the bars. *P < 0.05 vs NKX-Cre; ^#^*P* < 0.05 vs NKX-Cre; ^&^*P* < 0.05 vs NKX-Cre. (F) Masson’s trichrome staining of paraffin-embedded heart sections from NKX-Cre controls and the indicated PP1-deleted mice at 3 weeks of age. Scale bar, 50 μm.

Deletion of neither PP1 isoform from mouse heart led to any birth defect, suggesting PP1s are not involved in cardiac development [17]. To study whether PP1s have distinct functional roles in postnatal and adult hearts, we first compared the PP1 levels between neonatal and adult cells (**Fig. 4A–C**). The expression of PP1β was increased significantly in adult hearts, whereas PP1α and PP1γ maintained similar levels (**Fig. 4B,C**). Similarly from a real-time PCR analysis, PP1β expression was increased and significantly higher than PP1α and PP1γ in both cell types (myocytes and fibroblasts) of adult hearts (**Fig. 4A**). These data led us to hypothesize that PP1β plays an essential role in the mouse heart. Indeed as early as 3 weeks of age, PP1β fl/fl^NKX-Cre^ mice demonstrated increased interstitial fibrosis compared to other groups (**Fig. 4F**). Upon aging of these mice, deletion of either PP1 isoform led to reduced fractional shortening and left ventricular end-diastolic diameter (LVIDd) (**Fig. 4D,E**). These data suggest that PP1s are required for the normal cardiac function although the exact target of PP1β is yet to be identified.

**Figure 5.**
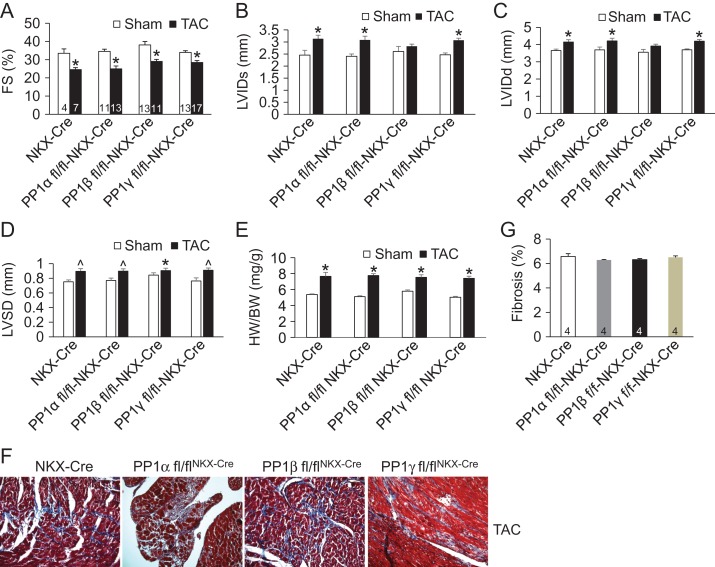
**Deletion of individual PP1 isoform does not influence cardiac stress responses** (A–D) Echocardiographic measurements of 2-month-old mice after 2 weeks of TAC. Mouse numbers for each group were indicated in the bars. *P < 0.05 vs sham (A–E). ^*P* < 0.05 vs sham. In D, *P < 0.05 vs NKX-Cre. (E) Measurements of heart/body weight (HW/BW) ratios in the indicated groups of mice after 2 weeks of TAC. *P < 0.05 vs sham. (F) Representative histological sections stained with Masson’s trichrome (shows fibrosis in blue) from TAC hearts of indicated groups of mice. (G) Quantification of interstitial fibrosis based on F. Mouse numbers for each group were indicated in the bars.

PP1s have been proposed as the therapeutic targets for heart disease treatment. It is still unknown whether PP1s are involved in pathological cardiac remodeling. To address this question, we subjected 8-week-old NKX-Cre control mice or mice with cardiac-specific deletion of each PP1 isoform to either a sham procedure or pressure overload stimulation by TAC. Cardiac function analysis by echocardiography demonstrated that after 2 weeks of TAC, fractional shortening was not significantly different among all groups of mice (**Fig. 5A**). Ventricular chamber dimensions (LVIDd and LVIDs) and septal width (LVSD) were all significantly increased after TAC except for PP1β fl/fl^NKX-Cre^ mice (**Fig. 5B–D**). It is possible that TAC stimulation did not lead to further ventricular dilation or growth because PP1β fl/fl^NKX-Cre^ mice already had disease at baseline (**Fig. 4F**). Similar cardiac hypertrophic growth after TAC was achieved among all groups (**Fig. 5E**). Moreover, histological analysis by Masson’s trichrome staining also demonstrated that deletion of individual PP1 isoform led to similar interstitial fibrosis (blue staining) after TAC compared to the NKX-Cre mice (**Fig. 5F,G**). These data suggest that PP1s are not involved in stress responses in the heart.

## Discussion

Our study further extends the complex function of PP1s in the heart due to their distinct subcellular localizations. Our major findings are following. (1) PP1β mainly localizes in the nucleus to regulate the phosphorylation of HDAC7 in addition to myofilament protein MLC2 [17]. Deletion of PP1β leads to cardiac dysfunction in mice, possibly due to the combined roles of PP1β in both the cytoplasm and nucleus. (2) Overexpression of PP1α reduces PLB phosphorylation, and deletion of PP1α results in reduced cardiac function in aging mice. (3) PP1γ is not linked to any substrate in this study, however its deletion also results in reduced cardiac function (**Fig. 4D,E**).

PP1s are serine/threonine protein phosphatases that dephosphorylate a variety of cellular substrates. Our study here showed that PP1α was localized in the cytoplasm to regulate the PLB phosphorylation, consistent with a previous study of PP1α in the heart using transgenic mouse approach [16]. However, deletion of endogenous PP1α from mouse heart did not alter PLB phosphorylation [17], indicating that PP1α might have a dose-effect on PLB phosphorylation. In this study, we further demonstrated that PP1β had no effect on PLB phosphorylation (**Fig. 2D–F**). Instead, PP1β was localized to the nucleus to bind and regulate HDAC7 phosphorylation [19] (**Fig. 3**). Although closely related to HDAC7, dephosphorylation of HDAC4 is influenced by PP2A [31,32]. HDAC5 phosphorylation is also influenced by PP2A [33]. However, our study didn’t provide further mechanism on how PP1β regulates HDAC7 transcriptional activity to control cardiac function. It is possible that dephosphorylation of HDAC7 by PP1β promotes its nuclear localization to suppress target gene expression as shown in thymocytes [19].

Congenital heart disease is one of the most common development disorders that affects ~8:1000 live births worldwide [34,35]. Missense variants in *PPP1Cb* gene were also implicated in congenital heart disease [36]. Although without any developmental defects upon deletion of PP1β from embryonic stage using NKX2.5-Cre, PP1β fl/fl^NKX-Cre^ mouse hearts demonstrated extensive interstitial fibrosis as early as 3 weeks of age and eventual reduction of cardiac function (**Fig. 4D,E**). Further investigations are needed to determine whether this phenotype is due to transcriptional activity of HDAC7 or combined effect of phosphorylation of MLC2 (**Fig. 2**). Interestingly, PP1α fl/fl^NKX-Cre^ and PP1γ fl/fl^NKX-Cre^ mice also demonstrated eventual reduction of cardiac function, which was possibly due to the compensational increase of other PP1s in these hearts [17].

Upon 2 weeks of pathological stimulation by TAC, mice with loss of either PP1 isoform developed similar hypertrophy and dysfunction compared to the controls, suggesting that PP1s are not involved in this process (**Fig. 5**). It will be interesting to extend the TAC stimulation to a longer period such as 14 weeks to assess whether PP1s are involved in the decompensation of the heart to failure. It is also possible that deletion of one isoform is compensated by increased levels of the other two isoforms, so the overall PP1 activity is not influenced [17]. However, even deletion of PP1β led to upregulation of both PP1α and PP1γ [17], PP1β fl/fl^NKX-Cre^ still developed reduction of cardiac function (**Fig. 4D–F**), suggesting that each PP1 isoform plays a unique role possibly due to their distinct subcellular localizations, which cannot be compensated by other PP1 isoforms.

In summary, our study provides further insights into the physiological role of cardiac PP1s which regulate different cellular substrates. One future direction is to identify the target genes regulated by PP1β to elucidate the pathology of PP1β fl/fl^NKX-Cre^ at 3 weeks of age. PP1γ apparently does not regulate PLB or HDAC7 phosphorylation, but deletion of it also influences the physiological cardiac function, suggesting that other potential substrates need to be verified or identified using our knockout models.
